# Delivery of essential surgery by family physicians

**DOI:** 10.2471/BLT.20.252056

**Published:** 2020-08-27

**Authors:** Eliana E Kim, David Araujo, Bruce Dahlman, Shivum Agarwal, Pratap Prasad, Walter Johnson, Kee B Park

**Affiliations:** a Program in Global Surgery and Social Change, Harvard Medical School, 641 Huntington Avenue, Boston, Massachusetts 02115, United States of America (USA).; b Ventura County Medical Center, Ventura, California, USA.; c Institute of Family Medicine, Nairobi, Kenya.; d Department of Family & Community Medicine, University of Texas Southwestern Medical Center, Dallas, USA.; e Institute of Medicine, Tribhuvan University Teaching Hospital, Kathmandu, Nepal.; f Emergency and Essential Surgical Care Programme, World Health Organization, Geneva, Switzerland.

## Abstract

Primary health care provides the framework for delivering the socially-informed, comprehensive and patient-centred care underlying robust health-care systems and is, therefore, central to achieving universal health coverage. Family physicians are best placed to embody primary health care’s dual focus on community and population health because they are often employed in rural or district hospitals with limited human resources, particularly a lack of specialists. Here we want to illustrate how additional training for family physicians, the key clinicians in primary care, can play a critical role in reducing disparities in access to surgical, obstetric and anaesthesia care in low- and middle-income countries and in rural or remote settings. Examples are given of how training programmes can be developed in low-resource settings to equip family physicians with life-saving surgical skills and of how family physicians in high-income countries can be trained in the surgical skills essential for working overseas in low-income settings. Policy-makers should promote surgical practice among family physicians by supporting family medicine programmes that include additional surgical skills training and by expanding opportunities and incentives for family physicians to serve in rural areas. Moreover, national surgical plans should include a primary health care strategy for surgical care and, globally, family physicians should be considered in discussions of surgical care. Finally, surgeons, anaesthesiologists, obstetricians and family physicians should be encouraged to collaborate in ensuring that all patients, regardless of place of residence, receive safe and timely surgical care.

## Résumé

Les soins de santé primaires établissent le cadre requis pour des soins complets adaptés aux patients, gages d'un système de santé solide. Ils jouent donc un rôle central dans la mise en place d'une couverture maladie universelle. Dans ce contexte, les médecins de famille sont les mieux placés pour incarner une double perspective, la santé communautaire et la santé des populations, car ils travaillent souvent dans des hôpitaux ruraux ou de district qui disposent de ressources humaines limitées, surtout en termes de spécialistes. Le présent document montre dans quelle mesure une formation complémentaire dédiée aux médecins de famille, acteurs clés des soins de santé primaires, peut avoir un impact décisif sur la diminution des inégalités d'accès aux interventions chirurgicales, obstétriques et anesthésiques dans les pays à faible et moyen revenu, ainsi que dans les milieux ruraux ou isolés. Il existe de nombreux exemples qui illustrent la façon dont les programmes de formation peuvent être mis en œuvre dans les endroits manquant de ressources afin que les médecins de famille acquièrent des compétences chirurgicales vitales, et qui indiquent comment les médecins de famille des pays à haut revenu peuvent apprendre des techniques de chirurgie essentielles pour travailler outre-mer ou dans des régions plus défavorisées. Les législateurs devraient promouvoir la pratique chirurgicale chez les médecins de famille en soutenant les programmes qui incluent une formation complémentaire aux techniques de chirurgie, et en multipliant les opportunités et sources de motivation pour que les médecins de famille exercent dans les zones rurales. Par ailleurs, les projets nationaux devraient comporter une stratégie de soins de santé primaires pour les actes chirurgicaux et, globalement, les médecins de famille devraient être pris en compte dans les discussions consacrées aux interventions chirurgicales. Enfin, chirurgiens, anesthésistes, obstétriciens et médecins de famille devraient être encouragés à collaborer pour s'assurer que tous les patients, quel que soit leur lieu de résidence, puissent bénéficier de soins chirurgicaux au moment opportun et en toute sécurité.

## Resumen

La atención primaria de salud establece el marco para la prestación de una atención socialmente informada, integral y centrada en el paciente, que es la base de los sistemas sólidos de atención sanitaria y, por lo tanto, es fundamental para lograr la cobertura sanitaria universal. Los médicos de familia son los que mejor pueden asumir el doble enfoque de la atención primaria en la salud de la comunidad y de la población porque trabajan con frecuencia en hospitales rurales o de distrito que tienen recursos humanos limitados, en especial por la falta de especialistas. En este documento, se pretende ilustrar cómo la capacitación adicional de los médicos de familia, quienes son los profesionales clínicos clave en la atención primaria, puede desempeñar una función esencial en la reducción de las desigualdades que existen para acceder a los procedimientos quirúrgicos, obstétricos y de anestesia en los países de ingresos bajos y medios y en los zonas rurales o remotas. También se dan ejemplos de cómo se pueden elaborar programas de capacitación en entornos de bajos recursos para preparar a los médicos de familia con técnicas quirúrgicas que salvan vidas y de cómo se puede capacitar a los médicos de familia de los países de altos ingresos sobre las técnicas quirúrgicas esenciales para que trabajen en el extranjero en entornos de bajos ingresos. Los responsables de formular políticas deben promover la práctica quirúrgica entre los médicos de familia mediante su apoyo a los programas de medicina familiar que incluyan una capacitación adicional sobre técnicas quirúrgicas, así como la ampliación de las oportunidades y de los incentivos para que los médicos de familia presten servicios en las zonas rurales. Además, los planes quirúrgicos nacionales deben incluir una estrategia de atención primaria de salud para la intervención quirúrgica y, a nivel mundial, los médicos de familia se deben tener en cuenta en los debates sobre la intervención quirúrgica. Por último, se debe alentar a los cirujanos, anestesiólogos, obstetras y médicos de familia a que colaboren para garantizar que todos los pacientes, sea cual sea su lugar de residencia, reciban servicios quirúrgicos seguros y oportunos.

## ملخص

توفر الرعاية الصحية الأولية الإطار المطلوب لتقديم الرعاية الشاملة، الواعية اجتماعياً، والتي تركز على المريض، والتي تعتمد على أنظمة الرعاية الصحية الفعالة، وبالتالي فهي مركزية لتحقيق التغطية الصحية الشاملة. يعتبر طبيب الأسرة في وضع أفضل لتجسيد التركيز الثنائي للرعاية الصحية الأولية على صحة المجتمع والسكان، وذلك لأنه غالبًا ما يعمل في مستشفيات ريفية أو محلية تعاني من موارد بشرية محدودة، وبخاصة نقص في المتخصصين. نريد هنا أن نوضح كيف يمكن للتدريب الإضافي لطبيب الأسرة، والأطباء السريريين الرئيسيين في الرعاية الأولية، أن يلعبوا دورًا حاسمًا في الحد من التفاوتات في الحصول على الرعاية في العمليات الجراحية، وعمليات التوليد، والتخدير في البلدان ذات الدخل المنخفض والمتوسط، وفي المناطق الريفية أو النائية. يتم تقديم أمثلة على كيفية تطوير برامج التدريب في الأماكن منخفضة الموارد، لتزويد طبيب الأسرة بالمهارات الجراحية المنقذة للحياة، وكيف يمكن تدريب طبيب الأسرة في البلدان ذات الدخل المرتفع على المهارات الجراحية الأساسية للعمل في الخارج في البلدان ذات الدخل المنخفض. يجب على واضعي السياسات الارتقاء بالممارسات الجراحية بين أطباء الأسرة من خلال دعم برامج الطب الأسري، والتي تشمل تدريبًا على المهارات الجراحية الإضافية، ومن خلال توسيع الفرص والحوافز لطبيب الأسرة ليعمل في المناطق الريفية. وبالإضافة إلى ذلك، فإنه يجب أن تشتمل الخطط الجراحية الوطنية على استراتيجية للرعاية الصحية الأولية خاصة بالرعاية الجراحية، كما يجب على الصعيد العالمي وضع طبيب الأسرة في الاعتبار في نقاشات الرعاية الجراحية. وفي النهاية، يجب تشجيع الجراحين، وأطباء التخدير، وأطباء التوليد، وأطباء الأسرة، على التعاون لضمان حصول جميع المرضى، بغض النظر عن أماكن إقامتهم، على رعاية جراحية آمنة، وفي الوقت المناسب.

## 摘要

初级卫生保健为在健全的卫生保健体系的基础上提供社会知情、全面的、以患者为中心的保健服务提供了框架，因此对实现全民健康覆盖至关重要。家庭医生最适合体现初级卫生保健对社区和人民卫生双重关注，因为他们通常受雇于人力资源有限的农村或地区医院，尤其缺乏专科医生。在此，我们想阐明对家庭医生（初级保健的主要临床医生）进行额外培训如何能够在缩小中低收入国家以及农村或偏远地区在获得外科、产科和麻醉护理的差距方面发挥关键作用。例如，如何在资源匮乏的环境下制定培训计划，以助家庭医生掌握挽救生命的手术技能，以及如何向高收入国家的家庭医生进行其在海外低收入国家工作所必需的外科技能培训。决策者应通过支持家庭医学计划（包括额外的外科技能培训）以及为家庭医生提供更多到农村地区服务的机会和激励措施，推动家庭医生的外科手术实践。此外，国家外科手术计划应涵盖外科手术护理的初级卫生保健战略，且全球各国在讨论外科手术治疗时都应将家庭医生考虑在内。最后，应鼓励外科医生、麻醉师、妇产科医生和家庭医生合作，以确保所有患者无论居住在哪，都可获得安全及时的手术护理。

## Резюме

Первичная медико-санитарная помощь обеспечивает основу для оказания социально информированной, комплексной и ориентированной на пациента помощи, лежащей в основе надежных систем здравоохранения, и поэтому играет центральную роль в достижении всеобщего охвата услугами здравоохранения. Семейные врачи наилучшим образом подходят для воплощения двойной ориентации первичной медико-санитарной помощи в вопросах здравоохранения сообщества и населения, поскольку они часто работают в сельских или районных больницах с ограниченными кадровыми ресурсами, в частности им недостает специалистов. Здесь мы хотим показать, как дополнительное обучение семейных врачей (основных врачей первичной медико-санитарной помощи) может сыграть решающую роль в сокращении неравенства доступа к хирургической, акушерской и анестезиологической помощи в странах с низким и средним уровнем доходов, а также в сельских или удаленных районах. Приведены примеры того, как можно разработать программы обучения в районах с низким уровнем ресурсов, чтобы вооружить семейных врачей хирургическими навыками, нужными для спасения жизни, и как семейные врачи в странах с высоким уровнем доходов могут обучаться хирургическим навыкам, необходимым для работы за границей в регионах с низким уровнем доходов. Лица, формирующие политику, должны продвигать хирургическую практику среди семейных врачей путем поддержки программ семейной медицины, которые включают дополнительное обучение хирургическим навыкам, а также путем расширения возможностей и стимулов для работы семейных врачей в сельских районах. Более того, национальные планы хирургической помощи должны включать стратегию первичной медико-санитарной помощи в отношении хирургической помощи, а в глобальном масштабе необходимо учитывать семейных врачей при обсуждении вопросов хирургической помощи. И наконец, следует поощрять к сотрудничеству хирургов, анестезиологов, акушеров и семейных врачей для обеспечения того, чтобы все пациенты, независимо от места проживания, получали безопасную и своевременную хирургическую помощь.

## Introduction

The World Health Organization (WHO), major global health funders and health-care organizations all regard primary health care as the cornerstone of universal health coverage (UHC). As it is based on the principle of equity, primary health care favours an approach to social and health policy development and implementation that prioritizes the collective and coordinated efforts of individuals, communities and stakeholders across all sectors.^1^^,^^2^ Primary care, an important element of primary health care, provides the foundations for a robust health-care system by delivering integrated health services that act as the first point of contact and that are continuous, comprehensive and coordinated – all characteristics known to improve health outcomes and patient satisfaction and to reduce costs.^3^^–^^6^ Moreover, in low- and middle-income countries, extensive coverage of primary health care has been associated with better population health.^7^ Consequently, strengthening primary health care and incorporating its core principles and components into health systems (including primary care) are essential steps towards achieving UHC globally.

Better access to surgical care is also critical for achieving UHC. In 2015, the World Health Assembly passed resolution WHA 68.15, which called for “strengthening emergency and essential surgical care and anaesthesia as a component of UHC.”^8^ Implementation of this resolution has been remarkable: by 2020, 37 countries were in various stages of developing a national surgical, obstetric and anaesthesia plan as part of their national health policy, strategy or plans. These surgical, obstetric and anaesthesia plans have introduced and streamlined processes for strengthening surgical provision in low- and middle-income countries by means of specific strategies in six main health-system domains: (i) infrastructure; (ii) workforce; (iii) service delivery; (iv) financing; (v) information management; and (vi) governance.^9^ In this way, national surgical, obstetric and anaesthesia plans are providing a systematic approach to addressing the unmet need for access to surgical care worldwide.

Unsurprisingly, most resources for implementing these plans are required for the infrastructure and workforce domains. In low- and middle-income countries, there are critical gaps in surgical care at district hospitals in rural settings with few resources due to a lack of specialist surgeons, obstetricians and anaesthesiologists. One potential solution is to incorporate the provision of essential surgical care into primary health care. In particular, we propose that family physicians, the main providers of primary care, could play a key role in delivering essential surgical, obstetric and anaesthesia services in rural areas where specialists are not available.

## Surgery in low-resource settings

Five billion people – roughly two thirds of the world’s population – lack access to safe, timely and affordable surgical, obstetric and anaesthesia care.^10^ Gaps in access occur predominantly in low-income settings: for example, only 6.5% of all surgery was performed in the poorest third of the world’s population in 2015.^10^ Over 143 million additional surgical procedures were needed that year to prevent premature death and disability,^10^ almost entirely among people in low-income areas with the greatest unmet need. In low- and middle-income countries, there was a severe shortage of the specialists needed to perform these essential procedures. Estimations show that there were only 0.7 surgical care practitioners per 100 000 population in low-income countries in 2015, which was a mere 1.2% of the corresponding figure for high-income countries and far below the recommended proportion of 20 to 40 licensed surgeons, obstetricians and anaesthesiologists per 100 000.^10^ In fact, better surgical, obstetric and anaesthesia care is a prerequisite for achieving the primary targets of sustainable development goal 3 (SDG 3) for good health and well-being, specifically: (i) reducing maternal mortality (SDG 3.1); (ii) preventing deaths in infants and children younger than 5 years (SDG 3.2); (iii) reducing premature deaths from noncommunicable diseases (SDG 3.4); and (iv) reducing deaths from road traffic accidents (SDG 3.6). In addition, surgical services are regarded as an integral component of UHC (SDG 3.8).^11^

A primary health care approach is vital for addressing the unmet need for essential surgical care in rural communities in low- and middle-income countries. Within the framework of primary health care, surgery could become part of an integrated approach to health-care delivery that is informed by local needs and priorities and that actively engages community stakeholders and community development organizations involved in public health.^12^ Family physicians could play an exceptionally important role. As champions of primary care, they are trained to provide one-stop comprehensive care (including essential surgical care) for patients of both genders, of all ages and with all pathological conditions throughout their entire lifetime.^13^ Moreover, the longitudinal and community-oriented nature of their practice places them in an ideal position to promote public health, as expressed in the Cairns consensus on rural generalist medicine.^14^ In fact, family physicians based in district hospitals have been regarded as central to the delivery and expansion of primary health care for rural populations.^15^^,^^16^ Thus, family physicians are the perfect candidates for providing surgical care within the framework of primary health care once they have undergone training in the surgical, obstetric and anaesthesia skills required locally.

In high-income countries, it is well established that training family physicians in additional surgical, obstetric or anaesthesia skills after their generalist training is very effective and is essential for those working in remote areas where it is daunting, expensive or risky to transfer patients to a central facility for routine procedures. Typically, family physicians spend an extra year training in one of these specialties. This approach has proved effective in the north-west territories of Canada, the outback of Australia and in underserved rural communities in the United States of America.^17^

In low- and middle-income countries, family physicians often perform a range of surgical procedures at district hospitals, such as emergency obstetric surgery and basic general surgery.^18^^,^^19^ Although it has been reported that these generalists can carry out selected surgical services efficiently, with good outcomes,^20^^–^^24^ insufficient data are available to determine whether the quality of the essential surgical care provided by nonspecialists matches that provided by specialists.^25^ As task-sharing and task-shifting become increasingly common in low-resource settings,^25^^,^^26^ it is important that generalists undergo the appropriate training and are equipped with the skills needed to ensure they can provide surgical care safely and effectively.

## Training generalists

A variety of training curricula and paradigms are available in low- and middle-income countries for teaching essential medical and surgical skills to generalist family physicians.^27^ In Nepal, for example, where over 90% of the population live in hilly, rural areas dominated by high-altitude mountains and plagued by natural disasters, the Nepalese general practice curriculum has been designed to tackle the problem of limited access to surgical care by training community-based physicians in the surgical skills needed in rural district hospitals.^28^ The first year of training covers general medicine, whereas the second year is dedicated entirely to building surgical skills, primarily in general surgery, orthopaedics, and obstetrics and gynaecology. Third-year general practitioner residents undergo training in emergency medicine and anaesthesia and are posted to rural regions. Residents commonly learn how to perform orthopaedic procedures for simple fractures and amputations, hernioplasties, caesarean sections, appendectomies and laparotomies for ectopic pregnancies.

Similarly in Kenya, while most citizens live in widely dispersed rural areas, most surgical specialists are located in large urban referral hospitals, leaving emergency surgical care in district and county hospitals to be dealt with by general physicians. The Kenyan Ministry of Health has designated family physicians as “the most appropriate person[nel] to respond to the challenges of the Kenyan health service delivery system” because of their ability to provide “continuous, comprehensive and cost-effective health care to individuals, families and communities.”^29^ Currently, five postgraduate programmes for family physicians in the country include training in essential surgical skills.^30^ For example, over a period of 4 years Kabarak University trains family medicine residents to independently diagnose and manage common, urgent and emergent problems that may require surgery, including paediatric, obstetric and gynaecological, orthopaedic and general surgery. The training enables family physicians to become competent in, for example, inguinal hernia repair, appendectomy, emergency haemorrhoidectomy, arthrotomy for septic arthritis, exploratory laparotomy, splenectomy, burr hole craniotomy and limb amputation.^30^

In high-income countries, family doctors are also trained to provide essential surgical and obstetric care for places with few resources. The Australian College of Rural and Remote Medicine programme,^31^ the Enhanced Surgical Skills programme of the College of Family Physicians of Canada and a variety of rurally-focused family medicine residencies in the United States all share the goal of equipping generalists with the skills needed for remote locations.^32^^–^^34^ For example, the Advanced Rural–Global Medicine and Surgery programme at John Peter Smith Hospital in Fort Worth, Texas has an expanded 4-year curriculum for family medicine residents that includes training in essential emergency and surgical (endoscopic, laparoscopic and open surgery) skills in areas such as neonatal and paediatric intensive care and obstetrics.^33^ The skills taught are based on consensus practice recommendations for low-resource settings from WHO (i.e. the primary surgical care package),^35^ the Council of Academic Family Medicine in the United States and other professional societies.^36^ There is also room in the programme to customize training to match the skills needed by individual trainees in their expected practice locations. Similarly, Ventura County Medical Center in Ventura, California has developed a training programme for family medicine residents that includes trauma resuscitation and stabilization, intraoperative skills in surgical techniques in a wide variety of procedures, primary caesarean section, high-risk obstetric care and critical care management. This training does not bring them into competition with surgical residents. In addition, a further year of training in either surgery or high-risk obstetrics is offered. Graduates have gone on to provide surgical care in places in low- and middle-income countries where there are no locally available surgeons.^37^ For decades, these programmes have enabled their graduates to develop the broad spectrum of medical, obstetric and surgical skills needed to serve successfully in low-resource, rural settings around the world.

## Improving surgical care

As these examples show, it is possible to train community-based physicians to provide medical and surgical care in rural, low-resource settings. However, much more needs to be done to popularize and institutionalize this practice. Evidence on surgical task-shifting and -sharing by nonspecialist physicians in low- and middle-income countries is growing. Documented experience in at least 29 countries in sub-Saharan Africa and in 10 countries in Asia demonstrate that task-sharing and -shifting are widely practiced out of necessity to bridge the gap in human resources needed for essential surgical care but that concrete policies supporting and formalizing this practice are largely absent.^25^ Often surgical training programmes for nonspecialist physicians do not have proper accreditation, sustainable funding or appropriate quality control measures.^25^^,^^38^ Generalists are also hindered from expanding their practice to include surgical services by role conflicts, the absence of a distinct professional identity and resistance from specialists due to shortfalls in policy and structural support.^25^

Policy recommendations based on current understanding of surgical task-sharing and -shifting in low- and middle-income countries call for the establishment of health governance structures that legitimize the nonspecialist provision of surgical care and that spearhead initiatives to reduce physician resistance to surgical training, to implement effective training systems and to enhance the career progression of nonspecialist physicians with surgical skills. Health ministries should: (i) formally define the scope of nonspecialists’ surgical practice; (ii) promote a distinct professional identity for nonspecialists with surgical skills; and (iii) adjust remuneration and benefits to match the expanded role of these nonspecialists.^25^ Models of surgical task-sharing and -shifting in Malawi and Mozambique have been cited as successful examples of government-endorsed training, accreditation and career development for the nonspecialists who provide the majority of essential surgery in rural areas and who achieve outcomes comparable to those of specialists.^39^^,^^40^ Reviewing and revising medical practice regulations has been suggested to accommodate new definitions of the role of nonspecialist physicians and to ensure their legal oversight and protection.^25^

The 2015 *Lancet* Commission on Global Surgery endorsed task-sharing as a strategy for expanding the surgical workforce and emphasized that task-sharing requires specialists and nonspecialists to collaborate and take joint responsibility for care.^10^ In contrast, task-shifting involves the transfer of surgical duties to nonspecialists without specialist oversight or shared responsibility. Although task-shifting is more common in low- and middle-income countries, task-sharing is considered safer and is preferred.^38^ Several of these countries responded to the *Lancet* Commission’s findings by creating national surgical, obstetric and anaesthesia plans that included task-sharing as a means of increasing human resources for surgical care. For example, the 2017 Zambian plan stipulated that safe and effective task-sharing should involve identifying existing task-sharing practices, defining the scope of practice of task-sharers and implementing outcome assessment tools.^41^ Similarly, the 2018 Tanzanian plan included the establishment of a task-sharing system as a strategic objective and considered task-sharers and -shifters as part of a collaborative entity for providing surgical care, designated as “allied health professionals”.^42^ The role of family physicians was not explicitly mentioned.

## Recommendations

We suggest that policy-makers should: (i) explicitly permit well-qualified, primary care specialists to practice surgery; (ii) expand family physician training programmes to include surgery; and (iii) incentivize young physicians to choose these programmes. Policy ambiguity about the scope of family physicians’ work can lead to confusion and produce resistance among specialists, especially those not willing to acknowledge that their specialty services may otherwise not be available in more rural districts. Emergency and essential surgical, obstetric and anaesthesia care are already being provided equitably by community-based, primary care physicians in many countries. This practice should be formally endorsed by the health ministries of low- and middle-income countries and the number of broad-spectrum, postgraduate training programmes should be expanded. In addition, the salaries of family physicians tasked with providing surgical care in rural areas should reflect their expanded range of practice. Labour market dynamics cannot be ignored; financial incentives are important for ensuring that skilled health professionals stay in rural areas where they are most needed instead of migrating to larger employment markets in cities or abroad.

In addition, national surgical, obstetric and anaesthesia plans should include policy and governance recommendations on the incorporation of surgical care into primary health care. The description of the workforce in these plans should be expanded to include not only surgeons, obstetricians and anaesthesiologists but also primary care providers, such as family physicians who have undergone postgraduate training in performing essential surgical procedures safely and effectively. The plans should also consider other types of health-care worker who are important for delivering surgical care, such as surgical officers, midwives and nurses, and their roles in relation to family physicians as surgical providers should be delineated. Moreover, training programmes should be defined by both surgical care specialists and community-based care providers, and national surgical, obstetric and anaesthesia plans should consider the estimated budget for these programmes. Finally, these plans should present strategies for ensuring essential surgical skills training is cost-effective and widely accessible nationally, for example by making use of existing medical schools and institutions and by designating them as central hubs for the postgraduate surgical training of family physicians who may be dispersed throughout the country.

As low-, middle- and high-income countries expand their training programmes for generalists, policy-makers should beware that programme development is an iterative process that, to be successful, requires financial budgeting for governance and research into outcomes and quality improvement measures. The quality of the education provided by programmes and trainees’ performance must be assessed through competency-based evaluations. Further, as demonstrated by the variety of existing programmes we have described, the tendency to create a one-size-fits-all approach should be avoided because training needs will vary according to local geographical conditions. Instead, programmes may benefit from adopting a hybrid approach that focuses on the mastery of fundamental medical and surgical knowledge, with added flexibility for region-specific skills training appropriate to the places where trainees will eventually practice.

Surgeons, obstetricians, anaesthesiologists and family physicians together need to develop a collaborative and mutually beneficial relationship that ensures that services are available for all citizens, including those living in geographically isolated areas. In this way, essential surgical care could be provided at district hospitals and specialists would be able to focus on more complex cases at referral hospitals where their more advanced skills can be supported by intensive care facilities. One way to foster such a relationship is for specialists to be key partners in training community-based family physicians in surgical skills, in providing active consultations and guidance, and in supporting family physicians’ continuing education. By enabling family physicians who have undergone postgraduate training to deliver as much front-line emergency and essential surgical care as possible, patients will benefit from timely access to surgical care, from a reduction in the number of referrals and from a more streamlined referral system when it is needed.

As primary health care plays a critical role in delivering UHC, primary care physicians must be equipped with the skills needed to provide the comprehensive medical and surgical care essential for ensuring that the health system is robust and resilient at the district level. In turn, this improvement in surgical care will accelerate progress towards achieving the sustainable development goals.
